# Muscle fatigue response of rotator cuff muscles in different postures

**DOI:** 10.1007/s00402-022-04650-8

**Published:** 2022-10-28

**Authors:** Lisanne Aranha, Charu Eapen, Vivek D. Patel, Ashish J. Prabhakar, Karthik Hariharan

**Affiliations:** 1 Department of Physiotherapy, Kasturba Medical College, Mangalore, Manipal Academy of Higher Education, Manipal, India; 2 School of Health and Rehabilitation Sciences, University of Pittsburgh, PA 15260 Pittsburgh, USA

**Keywords:** Rotator cuff muscles, Electromyography, Muscle fatigue, Posture

## Abstract

**Introduction:**

Muscle fatigue is a leading cause of rotator cuff (RC) pathologies. Scapular orientation affected by changes in the thoracic spine account for differences in body postures leading to altered RC muscle activation. This posture-related alteration in RC muscle activation and its fatigue response needs to be analyzed.

**Materials and methods:**

This study included 50 healthy shoulders with no coexisting spine pathologies. Raw data were recorded using electromyography sensors for RC muscles during two isometric maneuvers of abduction and external rotation, performed at 30% maximum voluntary contraction at 30°, 45°, and 90° arm elevation in sitting and standing. The raw data were analyzed in DataLITE^®^ software, and the mean power frequency (MPF) was extracted to analyze the fatigue response of RC muscles. The Wilcoxon signed-rank test and Kruskal–Wallis test with Bonferroni corrections analyzed fatigue differences between postures and various activities. *P* < 0.05 was considered significant for the results.

**Results:**

Supraspinatus muscle demonstrated significant fatigue at 90° of arm elevation in standing as compared to sitting (MPF −5.40: −5.41; *P* = 0.03) posture. Between the three elevation angles, all the RC muscles showed increased fatigue at 90° (MPF range −5.22 to −6.64). When compared between abduction and external rotation, only infraspinatus showed fatigue in external rotation (MPF range −5.42 to −6.08). Among all the three RC muscles, infraspinatus showed the maximum fatigue of MPF −6.64 when compared to supraspinatus −5.22 and teres minor −5.36.

**Conclusion:**

The findings indicate that alterations in the body postures and different elevation angles affect the RC muscles’ fatigue response.

## Introduction

The shoulder joint is a ball and socket type of synovial joint designed to move efficiently in all planes across a sizeable available range of movement. The dynamic stability needed for this highly mobile joint is made possible through joint concavity compression due to rotator cuff (RC) muscle contraction [1–4]. Thus, to maintain stability, RC muscles must efficiently contract to maintain joint congruency when forces act on the shoulder joint in the different positions of the elevation arc [4, 5]. The activity of RC muscles changes when the arm is held at different angles and in different planes [3, 6].

The orientation of the RC muscles makes them more vulnerable to injuries in overhead activities [7]. The arm elevation maneuvers are carried out efficiently in the scapular plane. In this plane, upward scapular rotation and glenohumeral joint movement can be carried out efficiently, which helps maintain an optimal length–tension relationship, sequentially reducing fatigue [3]. Fatigue, in turn, can produce scapular dyskinesis [8]. In addition, the scapular plane is ideal for achieving a full range of motion, as the inferior part of the joint capsule remains relatively lax. Several experimental studies have also established that as the RC muscles relax, they reduce the muscle fatigue felt by the arm during elevation in the scapular plane compared to the sagittal and coronal planes [3, 9]. Shoulder or glenohumeral (GH) elevation maneuvers are also affected by changes in the thorax and axial spine due to their effect on the orientation of the scapula [10, 11]. Body postures like upright sitting influence the degree of thoracic spine kyphosis compared to slouched sitting and standing [12, 13]. The postural change from sitting to standing increases thoracic kyphosis, which affects the scapular orientation [13, 14]. Therefore, scapular orientation affects the ability of the RC to generate force during muscular contractions [15].

RC muscle fatigue is the primary cause of shoulder pathologies due to its direct influence on humeral and scapular kinematics [16]. In addition, the RC muscles experience increased mechanical stress during work-related tasks at elevation angles above 60° [17]. Muscle fatigue, experienced due to repetitive activities, tends to have a cumulative trauma effect over time, resulting in musculoskeletal disorders [6]. Muscle fatigue is a complex phenomenon due to various biochemical and physiological changes, resulting in a decrease in the maximum power-generating capacity of the muscle [3, 18]. Continuous real-time fatigue monitoring is easy by surface electromyography (SEMG) which has strong validity and reliability [19, 20]. The degree of muscle fatigue estimated using the mean power frequency (MPF) is measured using SEMG [3, 21].

Appropriate functioning of the RC muscles is vital to ensure glenohumeral joint stability during arm elevation movements preventing superior translation of the humeral head [22]. However, there is currently no consensus in the literature concerning the dosage, timing, and postures to exercise the RC muscles [23]. Post-RC repairs, standard protocols for RC exercises include shoulder range of motion exercises in various postures without considering the influence of spinal posture on optimal muscle function or fatigue [24, 25]. Changes in scapular orientation associated with the thoracic rib cage and axial spine postures encourage exploring its influence on RC fatigue. Thus, assessing RC muscles and delivering the appropriate rehabilitative intervention will allow favorable treatment outcomes. Hence, this study set out to investigate two key aims. The primary objective was to evaluate, analyze and compare RC muscle fatigue in sitting and standing body postures. The secondary objectives were to explore and interpret the effects of different arm elevation angles and isometric maneuvers on the fatigue response of individual RC muscles.

## Methodology

### Approach

The study was conducted on healthy human participants. The supraspinatus, infraspinatus, and teres minor muscles were tested, as these muscles function primarily to stabilize the glenohumeral joint [26]. As the subscapularis muscle is deep, SEMG data cannot be obtained [27]. Thus, the subscapularis muscle was not included in this study. The MPF is commonly used and is the gold standard for assessing muscle fatigue [3, 28]. In SEMG studies, a shift in the MPF, directed towards lower coordinates or an increment in the power of low-frequency bands with a reduction in the power of high-frequency bands while performing isometric contraction indicates a state of muscle fatigue [3]. The MPF, obtained from raw EMG data, was used to analyze the fatigue in these muscles. Raw EMG data were collected during glenohumeral joint isometric maneuvers performed in sitting and standing.

### Participants

The study was conducted on 28 participants (50 non-painful shoulders). Participants were included in the study if they reported no complaints of existing shoulder pathology/pain in at least one shoulder within the last 6 months, no back pain, no history of shoulder/thoracic spine surgery, and no spinal deformities. The study included 22 females and 6 males with a mean age of 24.72 ± 9.01 years (18–54 years), a mean height of 159 ± 10.04 cm (144–180 cm), and a mean weight of 55.96 ± 10.98 kg (40–84 kg). The participants read and signed informed consent forms approved by the Scientific Committee and Institution Ethics Committee of KMC, Mangalore (IEC KMC MLR 11-19/583).

### Equipment

- Muscle strength testing apparatus
- MicroFET2^TM^ Muscle Test Dynamometer (Salt Lake City, Utah) is an accurate, digital, and portable device to test muscle strength. It consists of a Wireless MicroFET2^TM^ Digital handheld Muscle Tester, Curved transducer pad, and wall pack power supply. The device was placed over the proximal to the wrist. While participants performed abduction and external rotation of the glenohumeral joint, the isometric strength of the supraspinatus, infraspinatus, and teres minor muscles was measured [26, 27]. Participants were asked to generate maximum voluntary contraction (MVC).
- Electromyography apparatus
- The Biometrics DataLITE system (UK Biometrics Ltd Units 25–26 Nine Mile Point Ind. Est. Newport) consisted of the DataLITE Wireless SEMG sensors for the data collection. The SEMG sensors were placed over the patient interface using double-sided hypoallergenic and latex-free cut tapes. The SEMG sensors collected the raw data with 20–500 Hz band-pass filtering, >96 dB common-mode rejection ratio, and a Fast Fourier Transformation (FFT) algorithm (Kai et al. [3]; Motabar et al. [27]). The raw data were transferred to the Biometrics DataLITE Pioneer Wireless dongle, the interface between the sensors and the Biometrics DataLITE PC software.

### Data collection

Before each assessment, the participant underwent a brief postural awareness tutorial, including demonstrations of tasks and patient education on the effects of scapular position on muscle strength and output [13, 14]. In sitting, the participants were asked to flex their hips and knees at 90° each, and the corresponding instructions were “to sit as straight as possible, without leaning forwards or backward.” Participants were instructed to stand looking straight ahead with the feet facing forward and positioned shoulder-width apart in the standing posture. They were given the following instructions, “stand as straight as possible, without leaning forwards or backward.”

**Fig. 1:**
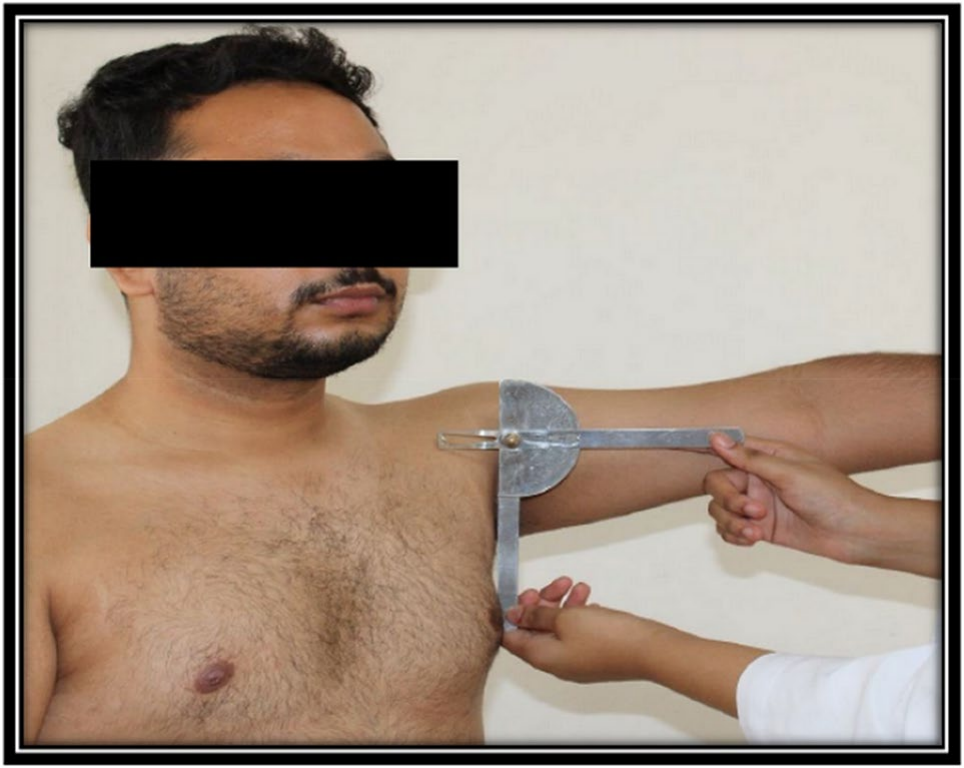
Position awareness of the participant at 900 of arm elevation

Participants were then made aware of the arm elevation angles of 30°, 45°, and 90° (Fig. 1). These elevation angles were selected based on evidence in the literature suggesting that RC muscles demonstrated high activity levels during the maneuvers performed at these elevation angles in the scapular plane [3, 26, 29]. Evidence in the literature shows that 30% MVC is the average level of exertion experienced at the workplace [27]. Participants were taught to maintain these positions at 30% MVC for 60 s or until the participants experienced fatigue [27, 30]. The participants performed two trials of each activity at the desired arm position. Raw EMG data were collected and recorded for supraspinatus, infraspinatus, and teres minor muscles.

### Procedure for SEMG electrode placement

The area for electrode placement was exposed and cleaned with alcohol wipes. The SEMG sensors were placed on the supraspinatus, infraspinatus, and the teres minor muscles parallel to the orientation of the muscle fibers. The EMG electrode was placed over the supraspinatus muscle, just above the middle of the spine of the scapula. For the infraspinatus muscles, the EMG electrode was placed over the muscle bulk approximately two finger breaths below the medial portion of the spine of the scapula. The distance between the acromion and the inferior angle was measured for teres minor muscle, and the EMG electrode was placed at approximately one-third of the distance measured from the acromion process [27].

### Procedure for muscle fatigue testing using EMG during sitting and standing (Fig. 2a, b)

**Fig. 2:**
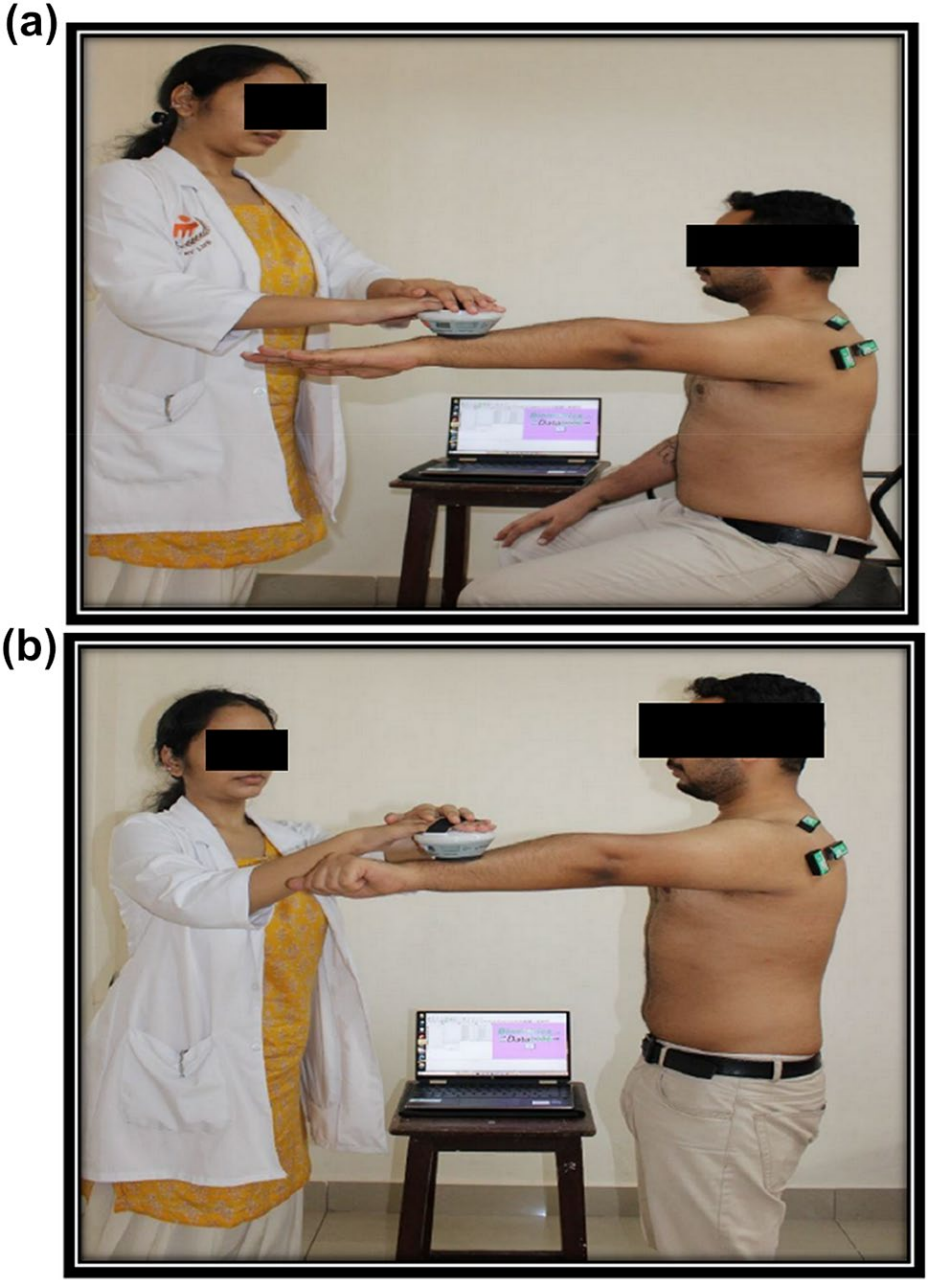
Muscle fatigue testing using EMG in sitting (**a**) and standing (**b**)

The participants were asked to maintain an upright sitting posture. The mean of 100% MVC trials during isometric arm abduction and external rotation performed at 30°, 45°, and 90° of arm elevation were measured using the MicroFET2^TM^ [3, 12, 20]. The 30% MVC was calculated from the mean of the 100% MVC values obtained. The 30% MVC shoulder excursions were monitored using the MicroFET2^TM^. The abduction and external rotation maneuvers at 30% MVC were performed at the three-arm elevation angles in both sitting and standing postures. Between the trials performed, a rest period of 1–2 min was given [3, 31].

### Data extraction

The raw data were isolated into 5 s intervals, and the MPF value for each interval was derived using the Biometrics Data LITE PC software. The MPF value was then entered into excel, and the data were analyzed using the Linest function of excel [3]. The final value for each raw data cycle was noted by calculating the statistics for a straight line using the Linest function.

### Data analysis

The final Linest function values were coded and entered into the Statistical Package for Social Sciences (SPSS) Version 25. The normality of the data was assessed using the Shapiro–Wilk test. However, the data did not follow the normal distribution; therefore, the Wilcoxon signed-rank test was applied to analyze and compare differences between postures and between isometric maneuvers. The Kruskal–Wallis and Bonferroni post hoc tests were performed to examine the relationship between RC muscle fatigue levels and arm elevation angles. A “*P*” value of <0.05 was considered statistically significant.

## Results

**Fig. 3:**
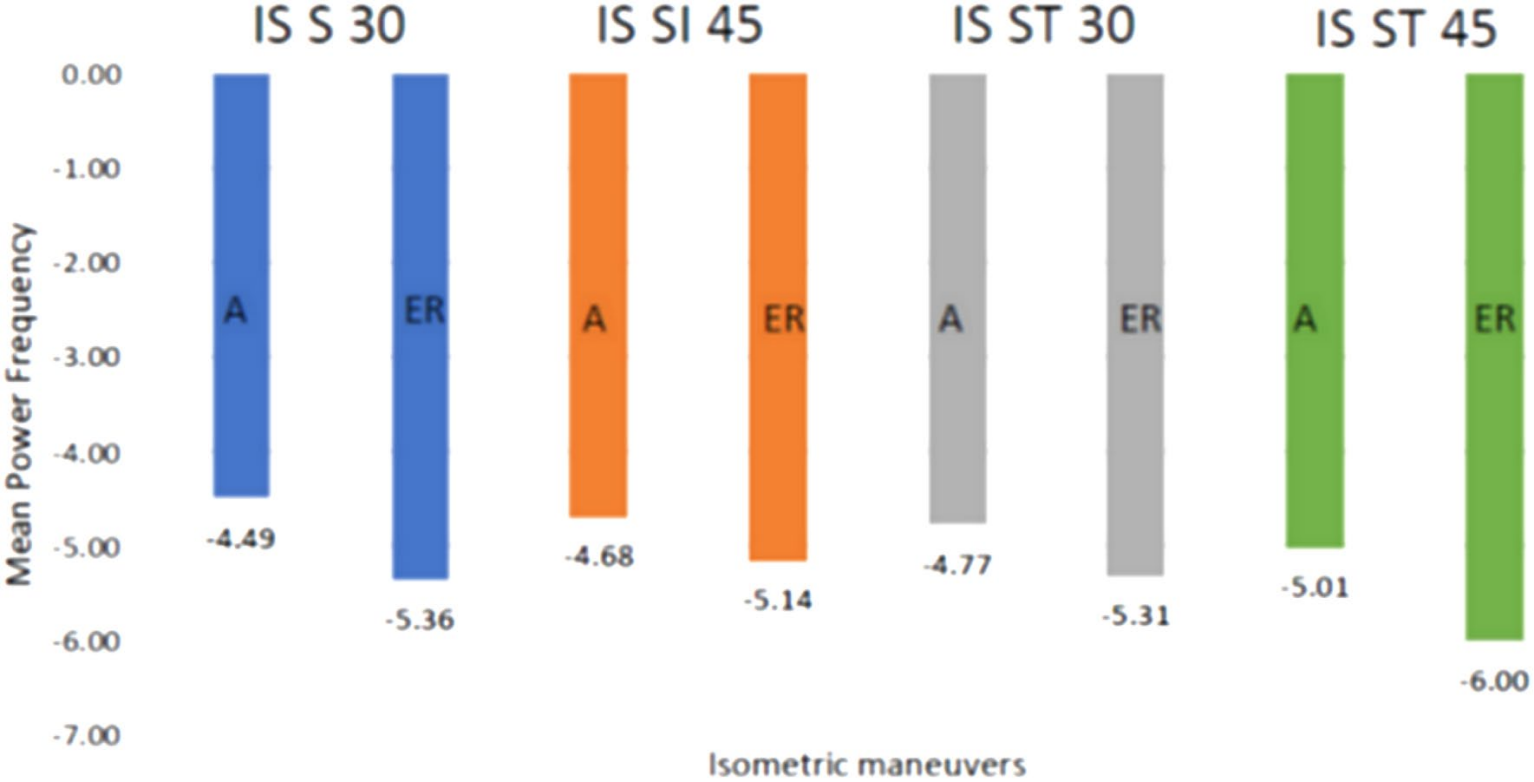
Average MPF of infraspinatus between the two maneuvers at 30° and 45° of elevation in both postures. *SI* sitting, *ST* standing, *A* abduction, *ER* external rotation, *IS* infraspinatus

**Fig. 4:**
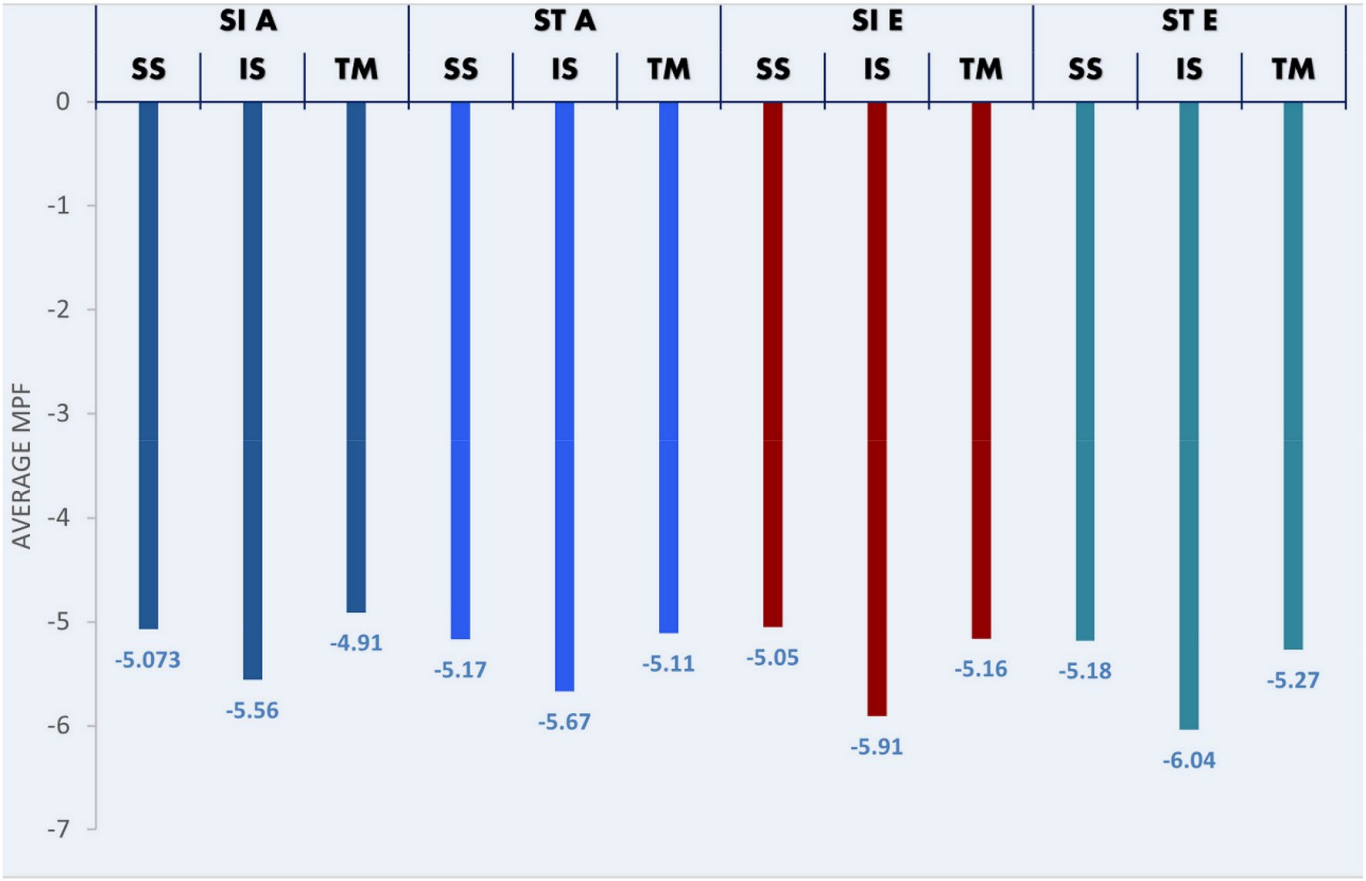
Average MPF of the RC muscles. *SI* sitting, *ST* standing, *A* abduction, *E* external rotation, *IS* infraspinatus, *SS* supraspinatus, *TM* teres minor

The first analysis comparing MPFs for fatigue response between two postures (sitting and standing) demonstrated no significant difference in all three RC muscles except for supraspinatus during an abduction at 90° (Table 1). When compared at the three elevation angles, in abduction, all three RC muscles demonstrated a significant difference in average MPF values in all three angles in either sitting or standing. However, for external rotation, supraspinatus and infraspinatus muscles showed no difference in fatigue between three angles of elevation positions in the sitting posture (Table 2). Table 3 further analyzes different elevation angles for abduction and external rotation movement (Table 3). If we compare the two maneuvers of abduction and external rotation, only the infraspinatus muscle displayed a significant difference in average MPF between the two maneuvers at only 30° and 45° of elevation in both postures; however, there was no difference observed for the other two muscles (Fig. 3). When the average MPFs were compared between the different RC muscles, significant differences were observed between the RC muscle activity with infraspinatus showing most fatigue (Fig. 4).

**Table 1:** Comparing average MPF of rotator cuff muscles between sitting and standing body postures

	Parameter			Median	IQR	Wilcoxon signed rank test *Z* value	*p*-value
IQR=	IS A 30°		Sit	−5.34	−6.26 to 2.63	−0.38	0.70
			Stand	−5.04	−6.4 to 4		
	IS A 45°		Sit	−5.26	−6.7 to 3.14	−0.63	0.53
			Stand	−5.63	−6.58 to 3.94		
	IS A 90°		Sit	−6.09	−7.15 to 3.88	−1.38	0.17
			Stand	−6.33	−7.44 to 4.68		
	IS E 30°		Sit	−5.42	−7.3 to 3.93	−0.27	0.81
			Stand	−5.61	−7.23 to 3.97		
	IS E 45°		Sit	−5.67	−7.81 to 1.64	−0.42	0.68
			Stand	−6.08	−8.02 to 4.4		
	IS E 90°		Sit	−6.64	−7.88 to 3.06	−1.84	0.07
			Stand	−6.43	−8.24 to 4.11		
	SS A 30°		Sit	−4.87	−5.89 to 2.17	−0.47	0.65
			Stand	−4.85	−5.79 to 3.29		
	SS A 45°		Sit	−4.95	−5.94 to 3.66	−0.13	0.90
			Stand	−5.24	−6.1 to 3.59		
	SS A 90°		Sit	−5.40	−6.53 to 3.83	−2.18	0.03*
			Stand	−5.41	−6.86 to 4.43		
	SS E 30°		Sit	−4.69	−6.19 to 2.97	−0.04	0.98
			Stand	−4.94	−6.23 to 3.05		
	SS E 45°		Sit	−5.25	−6.49 to 2.45	−0.22	0.82
			Stand	−4.99	−6.44 to 3.31		
	SS E 90°		Sit	−5.22	−6.67 to 3.02	−0.92	0.36
			Stand	−5.62	−6.56 to 2.82		
	TM A 30°		Sit	−4.57	−5.45 to 1.68	−1.31	0.19
			Stand	−4.75	−5.68 to 3.16		
	TM A 45°		Sit	−4.80	−5.84 to 3.34	−0.37	0.71
			Stand	−4.97	−6.65 to 2.81		
	TM A 90°		Sit	−5.35	−6.29 to 3.07	−0.25	0.81
			Stand	−5.62	−6.81 to 4.3		
	TM E 30°		Sit	−4.91	−5.83 to 3.45	−0.55	0.59
			Stand	−4.97	−5.56 to 3.4		
	TM E 45°		Sit	−5.22	−6.14 to 2.58	−0.89	0.37
			Stand	−5.17	−6.02 to 3.7		
	TM E 90°		Sit	−5.36	−6.7 to 3.78	−0.73	0.47
			Stand	−5.68	−6.52 to 3.74		

Inter-quartile range, *SS* supraspinatus, *IS* infraspinatus, *TM* teres minor, *E* isometric external rotation, *A* isometric abduction

^*^P < 0.05

**Table 2:** The average MPF of the rotator cuff muscles is compared between the elevation angles 30°, 45° and 90^o^

Parameter	Median	IQR	Kruskal–Wallis test value	*p*-value
IS SI A				
30°	−5.34	−6.26 to 2.63	12.52	0.002*
45°	−5.26	−6.7 to 3.14		
90°	−6.09	−7.15 to 3.88		
IS SI E				
30°	−5.42	−7.3 to 3.93	4.00	0.14
45°	−5.67	−7.81 to 1.64		
90°	−6.64	−7.88 to 3.06		
IS ST A				
30°	−5.04	−6.4 to 4	17.76	0.000*
45°	−5.63	−6.58 to 3.94		
90°	−6.33	−7.44 to 4.68		
IS ST E				
30°	−5.61	−7.23 to 3.97	13.32	0.001*
45°	−6.08	−8.02 to 4.4		
90°	−6.43	−8.24 to 4.11		
SS SI A				
30°	−4.87	−5.89 to 2.17	22.12	0.000*
45°	−4.95	−5.94 to 3.66		
90°	−5.40	−6.53 to 3.83		
SS SI E				
30°	−4.69	−6.19 to 2.97	0.84	0.66
45°	−5.25	−6.49 to 2.45		
90°	−5.22	−6.67 to 3.02		
SS ST A				
30°	−4.85	−5.79 to 3.29	14.88	0.001*
45°	−5.24	−6.1 to 3.59		
90°	−5.41	−6.86 to 4.43		
SS ST E				
30°	−4.94	−6.23 to 3.05	6.24	0.044*
45°	−4.99	−6.44 to 3.31		
90°	−5.62	−6.56 to 2.82		
TM SI A				
30°	−4.57	−5.45 to 1.68	23.04	0.000*
45°	−4.80	−5.84 to 3.34		
90°	−5.35	−6.29 to 3.07		
TM SI E				
30°	−4.91	−5.83 to 3.45	8.76	0.013*
45°	−5.22	−6.14 to 2.58		
90°	−5.36	−6.7 to 3.78		
TM ST A				
30°	−4.75	−5.68 to 3.16	16.84	0.000*
45°	−4.97	−6.65 to 2.81		
90°	−5.62	−6.81 to 4.3		
TM ST E				
30°	−4.97	−5.56 to 3.4	14.88	0.001*
45°	−5.17	−6.02 to 3.7		
90°	−5.68	−6.52 to 3.74		

*IQR*  Inter-quartile range, *SS*   supraspinatus, *IS*   infraspinatus, *TM*   teres minor, *E*   isometric external rotation, *A*   isometric abduction, *SI*  sit, ST = stand

^*^P < 0.05

**Table 3:** Post hoc comparisons between 30°, 45° and 90° of arm elevation

	*p*-value adjusted		
Parameter	At 45°–30°	At 90°–30°	At 90°–45°
IS SI A	0.34	0.003*	0.15
IS SI E	1.00	0.56	0.42
IS ST A	0.27	0.001*	0.07
IS ST E	0.91	0.002*	0.10
SS SI A	1.00	0.007*	0.001*
SS SI E	1.00	1.00	0.40
SS ST A	1.00	0.001*	0.00*
SS ST E	1.00	0.04*	0.07
TM SI A	0.02*	0.00*	0.02*
TM SI E	0.39	0.03*	1.00
TM ST A	0.26	0.001*	0.05
TM ST E	0.33	0.002*	0.003*

SS = supraspinatus, *IS* infraspinatus, *TM* teres minor, *E* isometric external rotation, *A* isometric abduction, *SI* sit, *ST* stand

^*^P < 0.05

## Discussion

This study examined the muscular fatigue of the RC muscles in various combinations of arm and body positions. The literature on RC muscles indicates that static exertions performed at 15–30% MVC produced a maximum drop in the EMG activity, indicating faster fatigue development in these muscles [27]. In this study, 30% MVC was assessed as EMG parameters related to muscle fatigue are more sensitive to changes in exertion when performed at the 15–30% MVC range [27]. The study’s primary aim was to assess and compare fatigue response for these muscles in seated and standing postures at varying angles of arm elevation commonly used in the rehabilitation of shoulder conditions [26]. The arm experiences minor fatigue in the scapular plane of elevation because of the relaxed orientation of the RC muscles; therefore, rehabilitation exercises are commonly performed in this plane [3, 9, 12]. This study chose the scapular plane to assess the RC muscles’ fatigue response. Since previous studies have shown that the muscles are active in different movements, the fatigue response was checked in isometric abduction and external rotation for the RC muscles [26, 32]. The primary aim was to see the fatigue response of all these muscles in postures commonly used in rehabilitation treatment programs for the RC muscles.

A decrease in the MPF towards lower frequencies indicates mechanical fatigue of the muscle [3]. The MPF analysis in the present study reveals that significant shifts in MPF were observed for the supraspinatus muscle in the standing posture with the arm abducted to 90°, indicating that this was the most ‘fatigable position’ for these muscles in standing. Interestingly, it was found that the MPF shifts during the isometric abduction maneuver for supraspinatus were significantly affected in the sitting posture in all three, 30°, 45°, and 90°, positions of arm elevation. It was concluded that the supraspinatus muscle went into considerable muscle fatigue in both sitting and standing, but the fatigue rate was more at 90° during isometric abduction in standing than in sitting. Supraspinatus has been associated with external rotation [33]. Our study found that the MPF shifts for the supraspinatus in external rotation were statistically significant in standing when compared at different angles, but it was not statistically significant when compared to MPF in sitting. This indicates that the supraspinatus muscles can experience fatigue in external rotation maneuvers performed in standing postures but not seated.

The sitting and standing postures did not affect the MPF shifts for the infraspinatus and teres minor muscles. The infraspinatus muscle is an external rotator [33]. It stabilizes the glenohumeral joint by directing the humeral head into the glenoid fossa [2]. Evidence in the literature suggests that the infraspinatus muscle plays a role in arm abduction [33–35]. Statistically significant differences in MPFs were observed at all the arm elevation angles during the external rotation and abduction maneuvers.

In addition, MPF differences during the isometric external rotation were more profound than in isometric abduction. The oblique part of the infraspinatus muscle fibers is morphologically advantaged due to its physical characteristics; it, therefore, plays a crucial role in the movements of the shoulder joint [34]. This asserts that significant muscle strength is generated in the infraspinatus muscle during arm maneuvers. The proportions of the mean fibers of the muscles influence the amount of muscle fatigue. Phasic muscles have an increased percentage of type II muscle fibers, fast-twitch fibers that fatigue quickly [36]. It has been found that the muscle fibers of all the RC muscles demonstrated a mixed pattern of type I and type II muscle fibers [3]. Evidence indicates that the infraspinatus and deltoid muscles contain increased type II fast-twitch fibers, causing them to fatigue faster during arm movements [37]. Our study showed more significant shifts in MPFs in the Infraspinatus muscle during the experimental movements than in supraspinatus and teres minor muscles, suggesting that the infraspinatus muscle experienced fatigue faster.

In addition, the teres minor muscle displayed significant MPF shifts at 30°, 45°, and 90°, with the most significant MPF shift at 90° arm elevation during the external rotation and abduction maneuvers. The teres minor muscle is an integral part of the posterior RC. The teres minor displays increased muscle activity to resist the excessive humeral head translation to provide glenohumeral joint stability [26].

The strength of this study was that all the RC muscles were checked in both external rotation and abduction, as they have been shown to contribute to both these movements. The individual RC muscles that were assessed demonstrated significant differences in the muscle fatigue responses with changing arm elevation angles. This information is useful when planning the exercises for the RC muscles, as fatigue leads to altered humeral and scapular kinetics [16]. On the other hand, the results of this study reveal that the preferred arm elevation angle for better recruitment of the RC muscles would be at 90° of elevation. This informs practice when prescribing exercises during later phases of rehabilitation when the primary aim involves strengthening the RC muscles [24]. The results of this study additionally posit that during the early phases of rehabilitation, the exercises may be performed at lower elevation angles to initiate a muscle contraction without adding excess stress on the RC. For the infraspinatus muscle, while muscle activity is present during both external rotation and abduction, the preferred movement for strength training exercises is external rotation on account of the fatigue levels revealed by the results of this study. Our results highlight the preferred angles and postures for workplace activities to avoid excessive strain on the RC muscles while executing arm elevation tasks. Workplace tasks at lower angles like 45° and 30° of elevation will exert less strain on the RC muscles.

The study had some limitations. There was a disparity in the number of males and females in this study with females being more. The subscapularis muscle could not be assessed as it is a deep muscle. Assessing the muscle activity of the subscapularis muscle using an indwelling type of fine wire electrodes was not feasible [27]. Another limitation was that fatigue was not checked at angles above 90° which may be involved in overhead activities and in sports. In addition, EMG activity could be checked in other postures like relaxed sitting or standing. Further research is necessary to study the effects of posture on shoulders with existing RC muscle pathologies and RC muscle activity in higher arcs of movement.

## Conclusion

The current study concluded that body postures significantly influence the muscle activity of the supraspinatus muscle. The supraspinatus muscle fatigued faster in standing during an abduction at 90° elevation. All the RC muscles displayed increased muscle fatigue at 90° of arm elevation. Exercises performed at lower angles like 45° and 30° of elevation will exert less strain on the RC muscles and can be used in the initial phases of rehabilitation or at the workplace. Between the external rotation and abduction movements, the infraspinatus muscle significantly increased fatigue levels during external rotation. Finally, the infraspinatus muscle showed marked muscle fatigue response among the RC muscles compared to the other two muscles.
